# Immunomodulatory Properties of Amniotic Membrane Derivatives and Their Potential in Regenerative Medicine

**DOI:** 10.1007/s11892-020-01316-w

**Published:** 2020-06-10

**Authors:** Charles-Henri Wassmer, Ekaterine Berishvili

**Affiliations:** 1 Cell Isolation and Transplantation Center, Department of Surgery, Geneva University Hospitals and University of Geneva, Geneva, Switzerland; 2 Faculty Diabetes Center, University of Geneva Medical Center, Geneva, Switzerland; 3 Institute of Medical Research, Ilia State University, Tbilisi, Georgia

**Keywords:** Amniotic membrane, Amniotic epithelial cells, Amniotic mesenchymal cells, Regenerative medicine, Immunomodulation

## Abstract

**Purpose of Review:**

During the last decades, the field of regenerative medicine has been rapidly evolving. Major progress has been made in the development of biological substitutes applying the principles of cell transplantation, material science, and bioengineering.

**Recent Findings:**

Among other sources, amniotic-derived products have been used for decades in various fields of medicine as a biomaterial for the wound care and tissue replacement. Moreover, human amniotic epithelial and mesenchymal cells have been intensively studied for their immunomodulatory capacities.

**Summary:**

Amniotic cells possess two major characteristics that have already been widely exploited. The first is their ability to modulate and suppress the innate and adaptive immunities, making them a true asset for chronic inflammatory disorders and for the induction of tolerance in transplantation models. The second is their multilineage differentiation capacity, offering a source of cells for tissue engineering. The latter combined with the use of amniotic membrane as a scaffold offers all components necessary to create an optimal environment for cell and tissue regeneration. This review summarizes beneficial properties of hAM and its derivatives and discusses their potential in regenerative medicine.

## Introduction

Regenerative medicine is a rapidly evolving, interdisciplinary field that aims to develop approaches for regeneration and repair of damaged tissues and organs. Major progress has been made in the development of biological substitutes applying the principles of cell transplantation, material science, and bioengineering [1]. Historically, the first attempt of the tissue replacement was skin grafting [2], establishing the basis for what would become plastic and reconstructive surgery. Since the turn of the millennium, the field of regenerative medicine has evolved rapidly along with the development of cell cryopreservation techniques, use of biocompatible materials, 3D bioprinting and generation of stem cell–derived tissue [3]. Mesenchymal stromal cells (MSC), especially from bone marrow (BM-MSC), embryonic stem cells (ESC), and, more recently, induced pluripotent stem cells (iPSC), have been intensively studied for their multilineage differentiation capacities, their anti-inflammatory and immunomodulatory properties, and are all candidate cell sources for regenerative medicine. They have demonstrated impressive capacities to improve outcomes of several inflammatory disorders [4]. However, in spite of significant advances, MSC-based therapy still faces several challenges: invasive extraction procedures to harvest the cells, loss of MSC potency during culture, clearance of the transplanted MSCs by the recipient and evidence of immuno-stimulatory properties of MSC under certain conditions [5, 6]. Furthermore, some studies reported a role for MSC in tumor growth [7–10]. Regarding ESC and iPSC, they have shown very promising results thanks to their ability for multilineage differentiation. However, they have also demonstrated a certain genetic instability rendering them vulnerable to mutation and tumorigenicity [11, 12]. Finally, achieving a sufficient cell mass for clinical application is a very labor-intensive endeavor in terms of logistics and equipment needed [13]. Altogether, stem cell–based therapy is not ready for large-scale clinical application and alternatives are required.

Human amniotic membrane (hAM) and its derivatives express similar characteristics and advantages as BM-MSC and exhibit a multilineage differentiation capacity. They are widely available, inexpensive, have limited ethical issues, and have no risk of tumorigenicity [14]. The hAM has been studied since many years and is used in the treatment of burns, skin defects, and corneal injuries [15, 16]. Because of their anti-inflammatory and immunomodulatory properties, human amniotic cells have been considered as valid candidates for cell therapy in several degenerative disorders [17–20].

In this article, we present an overview of the immunomodulatory properties of amniotic-derived tissues and their potential for application to regenerative medicine strategies.

## Placenta and Maternal Tolerance

The placenta is a temporary discoid-shaped organ forming barrier between fetal and maternal blood and representing the source of fetal antigens [21, 22]. The fetal part of the placenta originates from the blastocyst; in contrast, the maternal decidua is derived from the endometrium. The fetal surface of the organ consists of the chorionic plate covered by amniotic membrane and umbilical cord; the maternal surface of the placenta adjacent to endometrium is called the basal plate. Between these plates there is the intervillous space containing the placental cotyledons.

Pregnancy is a unique state in which a semi-allogenic fetus coexists inside the mother without being rejected by the maternal immune system [23]. This phenomenon of maternal tolerance is a complex process mediated by the restriction and modulation of leukocytes that permeate the maternal-fetal interface. Animal studies demonstrated significant reduction of T cell activation due to the indirect allorecognition of the fetus [24, 25]. Furthermore, low numbers of dendritic cells (DC) have been found in decidua, in spite of the natural killer (NK) cell abundance. This was explained by the absence of local lymphatic vasculature in the endometrium [26]. The effect of pregnancy and circulating fetal or placental antigens on T cell population has been also studied. It was shown that maternal T cells that can indirectly recognize the fetus are poorly primed and instead undergo clonal deletion [24]. Furthermore, studies on mice have demonstrated recruitment and induction of fetal-specific T regulatory (Treg) cells at the maternal-fetal interface, thus inducing tolerance to fetal antigens. Fetal-specific Treg cells are capable of persisting beyond parturition while maintaining their functionality [27, 28].

## Human Amniotic Membrane and Its Derivatives

**Fig. 1:**
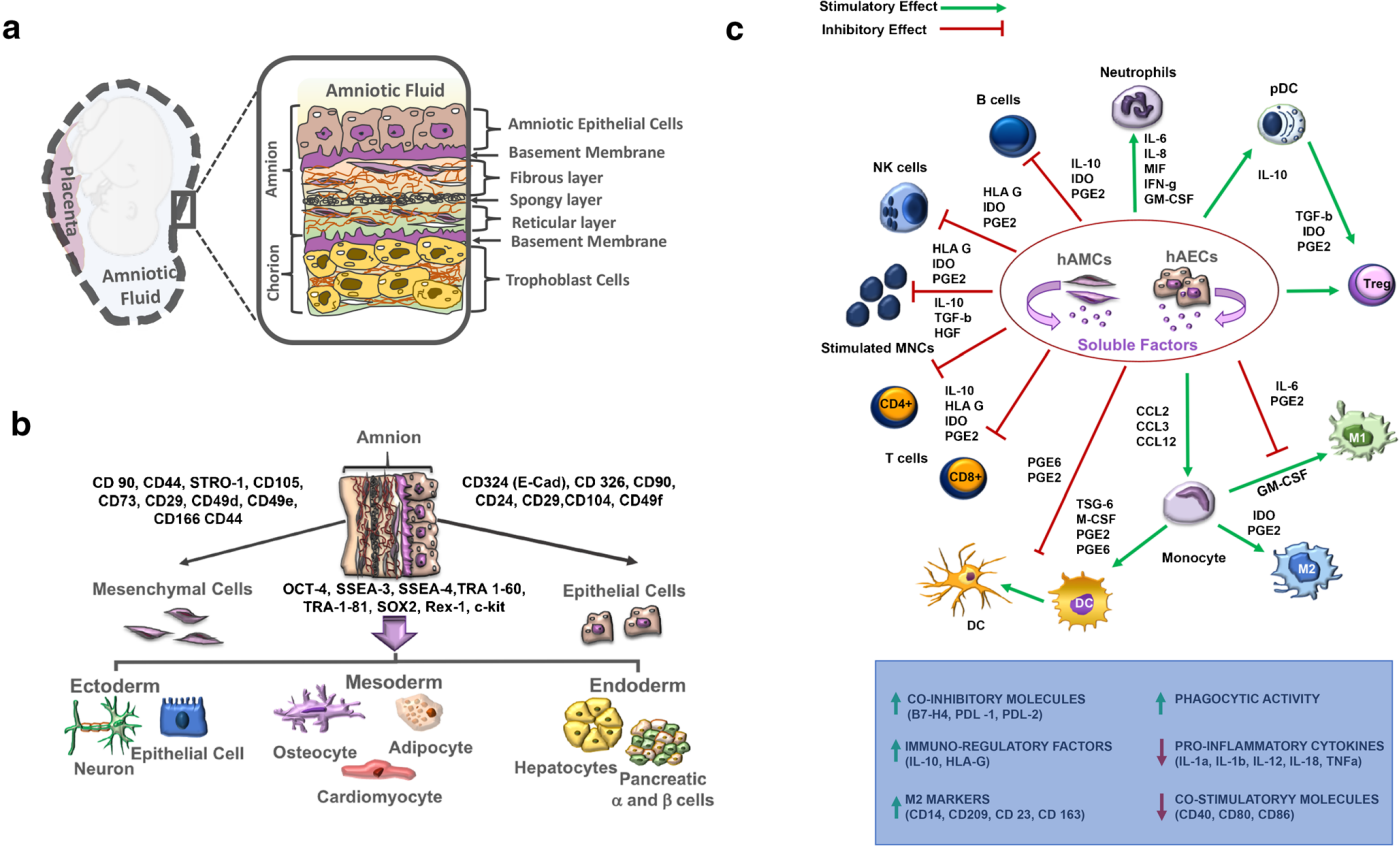
Amniotic membrane derivatives and their properties. **a** Graphical representation of amniotic membrane. hAM is made up of two main parts, the amniotic epithelium and the amniotic mesoderm, separated by a basement membrane. hAECs (brown) are found in amniotic epithelium adjacent to the first ECM layer, basement membrane (purple). The amniotic mesoderm consists of fibroblast (beige), spongy (black), and reticular (light green) layers containing hAMCs (purple). **b** Schematic diagram summarizing differentiation potential of hAECs and hAMCs into three embryonic germ layers, specifically ectoderm, mesoderm, and endoderm. **c** Immunosuppressive/immunomodulatory properties of hAECs and hAMCs. hACs are known to suppress the proliferation, inflammatory cytokine production, and differentiation of T cells. At the same time, they stimulate generation of Treg cells. Soluble factors secreted by hACs including PGE2, TGF-β, Fas-L, AFP, MIF, TRAIL, and HLA-G block dendritic cell and M1 macrophage differentiation and promote differentiation of monocytes into anti-inflammatory M2 phenotype. Moreover, hACs are known to be responsible for modulating host immune system, mainly through downregulation of TNF-α, IFN-γ, MCP-1, and IL-6 and upregulation of anti-inflammatory cytokines

The hAM is the innermost layer of the placenta and encloses the fetus in amniotic cavity. The hAM is avascular tissue composed of five layers: a monolayer of epithelial cells, an acellular basement membrane, a compact layer containing proteins of the extracellular matrix (ECM), a mesenchymal cell (hAMC) layer, and a spongy layer separating the amnion from the chorion (Fig. 1a) [29]. The compact layer and the fibroblast layer represent the amniotic mesoderm [30]. Among all amnion components, the hAM, the human amniotic epithelial cells (hAECs), and the human amniotic mesenchymal stem cells (hAMSC) are the most studied for their anti-inflammatory and immunomodulatory properties and will be the focus of this review.

### Human Amniotic Membrane

With its embryonic origin and protein contents, the hAM has long been considered to be a candidate as a biocompatible material for regenerative medicine. It has been used as a biological dressing for wounds healing for more than a century, since its first application by Davis in 1910, as a biologic material for the skin replacement [31]. Since then, hAM has been utilized for post-operative and post-traumatic skin defects, burn injuries, chronic ulcers, peritoneal, intra-oral and genital reconstruction, hip arthroplasty, tendon and nerve repair, dural defects, vascular reconstruction, and ophthalmologic disorders, mainly corneal defects [22, 32]. Interestingly, in contrast to skin allograft where immunosuppression is mandatory, hAM transplantation for skin or corneal defect has been performed without signs of rejection, in the absence of immunosuppression [22, 33]. Because it fails to induce an allogenic or xenogenic immunologic reaction, hAM has triggered great interest in transplantation and tissue engineering. This phenomenon results most likely from the combination of anti-inflammatory properties, low immunogenicity, and immunomodulatory properties. This favorable micro-environment is mainly created by the hAEC and hAMSC, notably by the secretion of growth factors, anti-inflammatory cytokines such as IL-10, and by expression of immunomodulatory proteins such as the non-classical MHC class I antigen HLA-G. In addition to the immunomodulatory properties of the cells residing in the hAM, its ECM has shown great promise as a biomaterial for tissue engineering thanks to its composition and properties. Several groups have decellularized amniotic membrane by established decellularization techniques, with which the cells are removed from tissues and organs using a combination of physical methods and chemical and biologic agents, only leaving an ECM scaffold from the original tissue. The decellularized hAM (dHAM) has shown prominent anti-inflammatory properties and provided mechanical protection and functional support for cell attachment, proliferation, and migration [34, 35]*.* It has been successfully used for peripheral nerve regeneration [36], neural differentiation [37], cartilage regeneration [38], as well as substrate for neo-vascularization development [39] and encapsulation. The extracellular matrix (ECM) of hAM is very similar to many other tissues of the body and the decellularization process does not alter its composition [40]. It is made of glycoproteins such as laminin, fibronectin, vitronectin, and nidogen, as well as a collagen types I, III, IV, V, and VI [41]. Furthermore, it contains fetal hyaluronic acid, which suppresses the expression of TGF-β1, β2, and β3, as well as TGF-receptor expression, providing an anti-fibrogenic effect [42]. The anti-inflammatory properties of hAM are believed to be both cytokine-mediated and mechanical. Solomon et al. observed a reduction of IL-1α and β expression and an increase of the anti-inflammatory cytokine IL-1RA in cells cultured on hAM after exposition to LPS [43]. A mechanical anti-inflammatory effect of hAM was observed in studies in vivo, where leucocytes trapped inside hAM stromal matrix rapidly entered apoptosis [44]. Finally, hAM possesses anti-microbial properties, making this an ideal biological dressing for wound healing. This effect is partially mechanical, offering protection against infectious organisms [45], but is also attributable to the presence of transferrin, bactricidin, β-lysin, lysozyme, and 7-S immunoglobulins in the amniotic fluid [46, 47]. Those molecules showed anti-bacterial effects against groups B and A streptococcus, *Enterococcus faecalis*, *Escherichia coli*, *Staphylococcus saprophyticus*, *Lactobacillus*, *Pseudomonas aeruginosa*, and *Acinetobacter* [48].

In summary, hAM is an inexpensive, widely available, biologically active and biocompatible tissue that can be banked for large utilization. This material is undoubtedly a major potential agent in the design of biological tissue engineering strategies.

### Amniotic Cells

hAEC and hAMSC can both be isolated from the hAM. hAECs reside on the first layer, directly in contact with the amniotic fluid and the fetus, while hAMSCs are found deeper, in the amniotic mesoderm. Freshly isolated hAECs usually express CD324 (E-Cad), CD326, CD9, CD24, CD29, CD104, and CD49f as well as the stem cell marker stage-specific embryonic antigens 3 and 4 (SSEA-3 and SSEA-4) and the tumor rejection antigen 1–60 and 1–81 (TRA-1-60 and TRA-1-81) (Fig. 1b). Finally, they also express Oct4, Sox2, Nanog, and Rex-1, members of the pluripotent stem cell transcription factor family [49, 50]. hAMSCs possess similarities with BM-MSCs and express mesenchymal markers such as CD90, CD44, STRO-1, and CD105 [51]. Like hAECs, they also express Oct4 and SSEA-4 [52]. Moreover, hAECs and hAMSCs have common cell surface markers (CD73, CD29, CD49d, CD49e, CD166, and CD44) and are both negative for the hematopoietic makers CD34 and CD45 and the monocytic marker CD14 [29]. By their potential to differentiate into the three germ lines (endoderm, mesoderm, and ectoderm) and their capacity to downregulate innate and modulate adaptive immunity, hAECs and hAMSCs have been studied and used in the treatment of inflammatory and immune-based disorders.

### Anti-Inflammatory Properties of Amniotic Cells

The downregulation of inflammation by amniotic cells (AC) is the result of their action on several key role players of the innate immunity. These suppressive effects have been demonstrated in cell-cell contact studies between ACs and immune cells, but also without contact, in a transwell model, or even only with conditioned medium (CM) from AC culture. For instance, neutrophil and macrophage migration is inhibited in vitro, as the result of migration inhibitor factor (MIF) secretion by hAECs [53]. A more recent in vivo study analyzed the ability of hAMSCs to improve corneal repair in a rabbit model and reported also a reduction of neutrophil migration to the injured site [52]. Furthermore, ACs have demonstrated the capacities to inhibit NK cell cytotoxicity by downregulating NK-activated receptors (NKp30, NKp44, NKp46, NKG2D, and CD69), and to reduce IFN-ɣ expression in a dose-dependent manner in vitro [54]. This suppressive activity was partially explained by an increased production of IL-10 and prostaglandin 2 (PGE_2_) by ACs when co-cultured with NK cells and was reversible when using anti-IL10 antibody or a specific PGE_2_ inhibitor. An immunosuppressive activity toward monocytes was also observed in this study. LPS-stimulated monocytes showed a reduction of pro-inflammatory cytokine (TNF-α and IL-6) production when cultured with ACs. Magatti et al. demonstrated that amniotic mesenchymal cells and their CM shift differentiation of monocytes toward an anti-inflammatory M2 phenotype [55••]. Furthermore, they observed a reduction of pro-inflammatory cytokine secretion (IL-1α, IL-1β, IL-12, IL-8, TNF-α, MIP1α, MIP1β, MIG, Rantes, and IP-10) by M2 macrophages, and an increased secretion of the anti-inflammatory cytokine IL-10. Finally, it was observed that M1 macrophages cultured with AC or their CM expressed less co-stimulatory proteins (CD80, CD86, and CD40) and induced a poor T cell response and a reduced number of IFN-ɣ-producing CD4^+^ T cells. They also demonstrated an increasing number of activated Tregs when purified T cells were co-cultured with either M1 macrophages exposed to CM during differentiation or M2 macrophages. The benefits of the shift toward the anti-inflammatory M2 phenotype was confirmed in several in vivo studies, for example, in liver fibrosis, lung fibrosis, and multiple sclerosis mouse models [56–59].

In summary, ACs strongly impair the development of an immune response by inhibiting neutrophil and macrophage migration, inducing M2 macrophage generation, reducing cytokine production by monocytes and NK cells and blocking the NK cytotoxicity.

### Immunomodulatory Properties of Amniotic Cells

It was thought for many years that one major characteristic of ACs was that they were not immunogenic and therefore under a state of immune tolerance. It has become clear that they are able to elicit immune responses, notably by expressing MHC class I (HLA-A,-B,-C) and II (HLA-DR), under certain conditions, for instance when cultured without serum or subjected to IFN-ɣ exposition [60]. This was demonstrated by in vitro and in vivo studies, in which an immune response was triggered by amniotic cells (Fig. 1c) [61, 62]. This means that the immune protection of ACs is the result of an active mechanism of suppression or modulation of the immune system.

In addition to downregulating the innate immune response, ACs have demonstrated their ability to suppress T cell proliferation in vitro in a dose-dependent manner [61–64]. Suppression was observed after T cell exposition to alloantigen in the presence of ACs, either after CD3/CD28 stimulation or in classic mixed lymphocyte reaction models. As for innate immunity suppression, the ability to strongly suppress T cell proliferation was observed with cell-cell contact, in a transwell system and with CM.

DCs are essential for the initiation of an immune response [65]. They present foreign or self-antigens to T cells, which can induce (i) CD4^+^ T cell clonal expansion and polarization in the Th1, Th2, or Th17 phenotypes, and (ii) CD8^+^ effector T cell proliferation and activation or, depending on co-stimulation factors, shift T cell differentiation toward Treg cells [66–68]. They also act on B and NK cells [69, 70] and are involved in the development of tolerance to self-antigens. Their interaction with immune cells in association with the environment will determine if the presented antigen will trigger a stimulatory of tolerogenic immune reaction. This critical role is obviously a target for cell-based therapy as tolerance can be induced by DC manipulation [71••]. It has been demonstrated that amniotic cells severely impair the function of monocyte-derived DCs by inhibiting their generation and maturation in vitro [53, 71••]. This phenomenon was observed not only in cell-to-cell contacts and transwell systems but also when monocytes were just exposed to CM. Although a direct cell contact is not necessary for this inhibition to occur, it was demonstrated that the negative effect on DC generation and function was stronger in cell-to-cell experiments. Furthermore, inhibition of DC generation seemed to decrease when hAECs with higher numbers of passages were used, most likely resulting from hAEC epithelial to mesenchymal transition [71••]. In addition to impair DC generation, it was observed that DCs exposed to ACs (in cell-to-cell or transwell systems) had significantly reduced capacities to stimulate CD4^+^ and CD8^+^ T cell proliferation. Finally, DCs exposed to ACs secreted higher level of anti-inflammatory cytokine IL-10 and reduced amounts of pro-inflammatory cytokines and chemokines (TNF-α, IL-12p70, IL-8, and MIP-1α) [71••].

One key element responsible for the immunomodulatory properties of ACs is the expression of the tolerogenic HLA-G [72]. This immunosuppressive molecule possesses 4 membrane-bound isoforms (HLA-G1, G2, G3, and G4) and 3 soluble isoforms (HLA-G5, G6, and G7). In addition to be present on hAECs, HLA-G expression can be induced on DCs when exposed to ACs during differentiation [73]. Furthermore, HLA-G expression is enhanced by IL-10 [74], IFN-α, -β, and -ɣ [75, 76]. The immunomodulatory properties of HLA-G result from the interaction with its corresponding receptors (ILT2, ILT4, and KIR2DL4) present on immune cells. While ILT4 is present on monocytes and DCs, ILT2 can be found on most immune cells (NK, CD4^+^, CD8^+^, B cells, monocytes, and DC). HLA-G interaction with DCs was studied in vitro and in vivo by Liang et al. and resulted in the inhibition of DC maturation and induced a differentiation toward the tolerogenic pathway [77]. Furthermore, DC function was altered by the reduction of MHC class II expression resulting in a decreased capacity to activate immune cells. It was also demonstrated that DCs exposed to HLA-G inhibited NK cell activation [78]. HLA-G interaction with T cells results in inhibition of proliferation, shift toward a Treg phenotype, CD8^+^ effector T cell inactivation, and apoptosis of previously activated CD8^+^ T cells [79]. With regard to B cells, HLA-G inhibits proliferation, immunoglobulin secretion, and chemotaxis. Finally, HLA-G also acts on innate immunity by suppressing NK cytotoxicity, through interaction with ILT2 and KIR2DL4 receptors, and by inhibition of ROS production and phagocytic capacity of neutrophils [80]. Those results were also observed in clinical studies, where HLA-G was associated with better allograft acceptance in transplanted patients [81, 82].

In addition to HLA-G, induction of tolerance by amniotic cells has been linked to their expression of the immune checkpoint proteins programmed death-ligands 1 and 2 (PD-L1 and PD-L2) [83]. In the placenta, these molecules are present on hAMSCs and in the syncytiotrophoblasts, but they can be induced in hAECs by IFN-ɣ exposition [60]. The interaction of PD-L1 and PD-L2 with their receptors will inhibit inflammatory cytokine secretion (IFN-ɣ, TNF-α, IL-2), and suppress T cell differentiation and proliferation [84].

In summary, ACs are able to block the initiation of an immune reaction by strongly altering the APC role of DCs. Furthermore, they inhibit CD4^+^ and CD8^+^ T cell proliferation, T cell cytotoxicity, and induce the development and expansion of the Treg cell population. For these reasons, ACs have been implicated in numerous inflammatory and immune disease models. They also represent an interesting source of cells in regenerative medicine thanks to the anti-inflammatory and immunomodulatory properties they are able to confer.

## Application in Regenerative Medicine

After having described the numerous advantages of amniotic membrane derivatives, this review will address their potential application in regenerative medicine, according to types of disorders to be treated. There are actually more than 180 ongoing or completed clinical trials registered worldwide, in which amniotic membrane derivatives are utilized, in almost every field of medicine: ophthalmology, plastic surgery, dermatology, cardiology, neurology, urology, diabetology, nephrology, pneumology, hepatology, transplantation, dental surgery, gynecology, orthopedic surgery, and ENT (ear nose throat).

### Amniotic Membrane Derivatives as Wound Dressing

As previously mentioned, amniotic membrane derivatives have been used for decades as wound dressings for skin burns [85], chronic ulcers of arterial, venous, or diabetic origin [86], in abdominal wall [87] and dural defects [88] and in corneal injuries (traumatic or chemical) [52]. The benefit provided was a mechanical protection, in association with anti-fibrogenic, anti-inflammatory, and anti-microbial properties. In vitro and in vivo results reported an increased cell migration and epithelization resulting in accelerated wound healing. HAM has also been studied in orthopedic surgery where it showed capacities to prevent the formation of adhesions in tendon repair [89]. Finally, hAM wrapped around nerve autografts in animal models of nerve injury was able to prevent perineural scarring and adhesion, increasing functional recovery [36, 90]. However, this improvement was only observed in short-term outcome, possibly because of degradation of hAM after a few weeks.

### Tissue Engineering and Cell-Based Therapy

ACs have been studied in several inflammatory diseases because of their anti-inflammatory properties but also for their potential to differentiate into many cell types, inducing tissue regeneration. Lung fibrosis can be idiopathic or secondary to chemical or physical insults. Several studies have demonstrated the benefit of hAEC transplantation in the bleomycin-induced mouse model. In addition to reduce fibrosis, inflammatory cell infiltration, and cytokine production, hAEC showed the capacity to differentiate into alveolar epithelial cells in vitro and in vivo, making them a promising material for lung regeneration [57, 91].

Similar results were observed in a liver fibrosis mouse model in which HAEC transplanted intravenously decreased fibrosis, inflammation, and apoptosis [92]. The same results were observed in a recent study, using a murine model of steatohepatitis [19]. The improvement was observed by injection of hAECs but also only with their CM. Furthermore, hAECs have been successfully differentiated into hepatocytes and cholangiocytes, in vitro and in vivo, improving tissue recovery [93, 94]. It is noteworthy that hAECs were able to improve liver function in those studies without being rejected, despite the fact that animals were immunocompetent.

HAMSCs have also demonstrated their capacity to improve kidney function in a kidney fibrosis rat model by reducing collagen deposition, inflammatory cell infiltration, and apoptosis [95].

Type 1 diabetes is a worldwide health issue. Replacing the lost β cells has been successfully performed by pancreas and islet transplantation. However, the scarcity of organ donors and the need for lifelong immunosuppression are the two major obstacles to generalize these therapies to the whole type diabetes patient population. ACs have been identified as a robust option to overcome these issues, by using two types of strategies. The first is to improve islet survival and engraftment by co-transplanting them with ACs as organoids. This has been successfully achieved by our group and others. Islets co-cultured with hAECs showed a better survival in hypoxic conditions and an increased functional potency compared with unmodified islets [96]. These results have been confirmed in immunodeficient [72, 96] and xenogeneic mouse models [97•]. In addition to improved glycemic control in vivo, histological assessments have demonstrated an increased vascularization of the grafts. The second is to use the stemness characteristics of amniotic cells as a source for differentiation into insulin-producing cells. hAECs have been successfully differentiated into cells with a β cell phenotype, with the capacity to control glycemia in streptozotocin-induced diabetic mice [98, 99].

Cell therapy using ACs, among other cell types, have also been used in cerebral ischemic stroke models and showed promising results by improving tissue recovery and reducing the volume on infarcted tissue [100–102].

DHAM has also been successfully used as a scaffold where adipose-derived mesenchymal stem cells were seeded and the whole construct was used in a myocardial infarction rat models. Regeneration of cardiomyocytes and reduced fibrosis were observed [103].

Finally, amniotic cells also have the capacity to differentiate into chondrocytes, osteocytes, myocytes, and adipocytes and have been studied for osteochondral disorders [104, 105].

The anti-inflammatory and immunomodulatory capacities of ACs have also been evaluated in T cell-mediated disease such as autoimmune disorders, and allo-rejection in transplantation models. Intravenous injection of hAECs improved clinical outcomes in an experimental model of autoimmune encephalomyelitis, used for the study of multiple sclerosis [59]. Cellular infiltration and demyelination were significantly reduced in the animals treated with hAECs and a T cell shift toward the Th2 phenotype was observed. HAEC injection in a mouse allogeneic skin transplantation model improved engraftment and survival, arguably by tolerance induction [106]. This was also demonstrated in a kidney graft model in the rat, in which amniotic cells inhibited acute rejection, inflammatory cell infiltration, and supported graft function [107].

## Conclusions

In this review, we have described the unique characteristics of amniotic membrane derivatives, making them an attractive resource for application to a large number of strategies in regenerative medicine. ACs possess two major characteristics that have already been widely exploited. The first is their ability to modulate or even suppress the innate and adaptive immunity, making them a true asset for chronic inflammatory disorders and for the induction of tolerance in autoimmune and transplantation models. The second is their multilineage differentiation capacity, offering a source of cells for tissue engineering. The latter, combined with the use of hAM as a scaffold, offers all components necessary to create an optimal environment for cell and tissue regeneration.

One limitation of amniotic cells is the progressive loss of their beneficial capacities over culture passages. This has been demonstrated for example in experiments studying liver and lung fibrosis where the anti-inflammatory effects exerted by hAECs were markedly reduced after several passages. It was partially explained by an increased expression and secretion of MCP-1 (monocyte chemoattractant protein-1) by hAEC after 5 passages, and resulted in greater infiltration by inflammatory cells [64, 91]. Magatti et al. also observed a reduced capacity to inhibit monocyte-derived DC differentiation with passaged amniotic cells. The difference through passages was more obvious with hAECs [71••]. This differentiation through passages impairs the capacity to expand those cells and should be overcome in order to obtain a sufficient cell amount for clinical use.

It has been demonstrated that the expression of immunomodulatory proteins by amniotic cells differs depending on their location on the amniotic membrane. More precisely, hAM can be divided in two regions, the placental and reflected areas [108]. Differences in morphology and functional activity have been observed between those areas, notably in the expression of HLA-G and TGF-β by the amniotic cells [109]. Finally, there are conflicting reports in the literature concerning amniotic cell expression of classical MHC class I and class II, as well as their capacity to induce an immune reaction. Some studies report low or only inducible expression of HLA-A, B, C by INF-ɣ [57, 110, 111], while others observed a clear constitutional expression of these molecules [63, 71••, 112]. This may mostly be indicative of a high level of cell heterogeneity between placentas, but also between placenta regions, and amount of cell passages. It is therefore important to always assess the expression and the suppressive capacities of the ACs before using them.

Much work still has to be done in order to reduce heterogeneity and to improve the immunosuppressive activity over the time. The impact of culture conditions and medium components on cell surface markers should be analyzed carefully. Selection of cells by cell sorting based on their phenotypes before expansion can increase the homogeneity. Finally, gene editing by clustered regularly interspaced short palindromic repeats (CRISPR-Cas9) can offer a novel and accurate mechanism by which these cells can be manipulated in order to extract their best capacities and to render them the most adapted cell source for regenerative medicine.
